# Genome-wide identification of oil biosynthesis-related long non-coding RNAs in allopolyploid *Brassica napus*

**DOI:** 10.1186/s12864-018-5117-8

**Published:** 2018-10-12

**Authors:** Enhui Shen, Xintian Zhu, Shuijin Hua, Hongyu Chen, Chuyu Ye, Longhua Zhou, Qing Liu, Qian-Hao Zhu, Longjiang Fan, Xi Chen

**Affiliations:** 1 Institute of Crop Sciences & Institute of Bioinformatics, College of Agriculture and Biotechnology, Zhejiang University, Hangzhou, 310058 China; 2 Institute of Crop and Utilization of Nuclear Technology, Zhejiang Academy of Agricultural Sciences, Hangzhou, 310021 China; 3 CSIRO Agriculture and Food, GPO Box 1700, Canberra, ACT 2601 Australia

**Keywords:** *Brassica napus*, lncRNA, Coexpression, Oil biosynthesis, Conservation

## Abstract

**Background:**

Long noncoding RNAs (lncRNAs) are transcripts longer than 200 bp that do not encode proteins but nonetheless have been shown to play important roles in various biological processes in plants. *Brassica napus* is an important seed oil crop worldwide and the target of many genetic improvement activities. To understand better the function of lncRNAs in regulating plant metabolic activities, we carried out a genome-wide lncRNA identification of lncRNAs in *Brassica napus* with a focus on lncRNAs involved in lipid metabolism. Twenty ribosomal RNA depleted strand specific RNA-seq (ssRNA-seq) datasets were generatred using RNAs isolated from *B. napus* seeds at four developmental stages. For comparison we also included 30 publically available RNA-seq datasets generated from poly(A) enriched mRNAs isolated from from various *Brassica napus* tissues in our analysis.

**Results:**

A total of 8905 lncRNA loci were identified, including 7100 long intergenic noncoding RNA (lincRNA) loci and 1805 loci generating long noncoding natural antisense transcript (lncNAT). Many lncRNAs were identified only in the ssRNA-seq and poly(A) RNA-seq dataset, suggesting that *B. napus* has a large lncRNA repertoire and it is necessary to use libraries prepared from different tissues and developmental stages as well as different library preparation approaches to capture the whole spectrum of lncRNAs. Analysis of coexpression networks revealed that among the regulatory modules are networks containing lncRNAs and protein-coding genes related to oil biosynthesis indicating a possible role of lncRNAs in the control of lipid metabolism. One such example is that several lncRNAs are potential regulators of *BnaC08g11970D* that encodes oleosin1, a protein found in oil bodies and involved in seed lipid accumulation. We also observed that the expression levels of *B. napus* lncRNAs is positively correlated with their conservation levels.

**Conclusions:**

We demonstrated that the *B. napus* genome has a large number of lncRNA and that these lncRNAs are expressed broadly across many developmental times and in different tissue types. We also provide evidence indicating that specific lncRNAs appear to be important regulators of lipid biosynthesis forming regulatory networks with transcripts involved in lipid biosynthesis. We also provide evidence that these lncRNAs are conserved in other species of the Brassicaceae family.

**Electronic supplementary material:**

The online version of this article (10.1186/s12864-018-5117-8) contains supplementary material, which is available to authorized users.

## Background

Non-coding RNAs (ncRNAs) are transcripts without a clear coding protein capacity found in the transcriptomes of plants and animals at an increasing frequency in recent years [1]. The role of ncRNAs is still not fully known but has been suggested to be involved in regulation of gene expression, translation, cell-cycle progression and other cellular functions [2, 3]. There are diverse kinds of ncRNAs that have been generally grouped into housekeeping and regulatory ncRNAs. The housekeeping ncRNAs include transfer RNAs (tRNAs), small nuclear RNAs (snRNAs), small nucleolar RNAs (snoRNAs) and ribosomal RNAs (rRNAs). The regulatory ncRNAs fall into two subclasses in plants. One type is the small RNAs (sRNAs), including microRNAs (miRNAs) and small interfering RNAs (siRNAs) with a size of 20–24 nucleotides (nt). sRNAs achieve their functions via two main mechanisms: transcriptional gene silencing (TGS) and posttranscriptional gene silencing (PTGS). Another type is long non-coding RNAs (lncRNAs) with a size defined as longer than 200 nt. LncRNAs have been shown to function in response to a wide range of biotic and abiotic stresses in plants [4–7]. LncRNAs are grouped according to their genomic location and orientation relative to their nearby protein-coding genes. Long intergenic noncoding RNAs (lincRNAs) locate in the interval between two genes. Long noncoding natural antisense transcripts (lncNATs) are those overlapping with protein coding genes in the opposite orientation. Long intronic noncoding RNAs are generated from intron of other transcripts and sense lncRNAs are those partially overlapping with protein coding genes on the same strand [8, 9]. LncRNAs are usually lowly expressed and tissue-specific [10]. Plant lncRNAs have been shown to be involved in transcriptional gene silencing, gene expression regulation, chromatin structure remodeling and other epigenetic mechanisms [11–15].

With the development of high throughput sequencing technologies and the ability to generate large numbers of transcriptomes, there has been an ever increasing number of lncRNAs identified in plants including Arabidopsis [16–22], rice [11, 23–26], maize [27–29], wheat [30, 31], and cotton [32–34]. Some lncRNA candidates have been identified in *B. napus* [35] and *B. rapa*, one of the two ancestors of *B. napus* [36, 37] and in synthesized *Brassica* hexaploids, but to date at genome-wide identification of lncRNAs in *B. napus* has not been reported.

*B. napus*, also known as oilseed rape, is second only to soybean as an oil crop with a world production of over 60 million tons [38]. *B. napus* is an allotetraploid (A_n_A_n_C_n_C_n_) evolved from a spontaneous hybridization event between *B. rapa* (A_r_A_r_) and *B. oleracea* (C_o_C_o_) about 7500 to 12,500 years ago [39]. With the availability of the *B. napus* genome sequence [39], it is now possible to identify and characterize lncRNAs at the whole-genome level in this important oil crop.

Oil biosynthesis is one of the key biological processes in *B. napus* and a major focus of much experimental research [40, 41]. Up to now, a role of ncRNAs in lipid and fatty acid metabolism in *B. napus* has only been investigated to a very limited extent [42–44]. Some miRNAs were found to be differentially expressed in cultivars with different seed oil content [43]. Shen et al. (2015) found that 122 lipid-related genes are potentially regulated by 158 miRNAs. Recently, Wang et al. (2017) further showed that 11 miRNAs may have regulatory relationships with 12 lipid-related genes.

To further investigate the possible role of lncRNAs in the control of oil biosynthesis in *B. napus*, we have conducted a comprehensive analysis of lncRNAs at multiple stages of seed development. We also collected 30 publically available RNA-seq datasets generated from different tissues of *B. napus*.We show that the *Brassica napus* genome contains a large number of tissue and developmental stage specific lncRNAs and that some of these form part of regulatory networks specifically involved in the control lipid biosynthesis. We also show that some of these regulatory lncRNAs are conserved in other species of the Brassicaceae family, including the two progenitors (*B. rapa* and *B. oleracea*) of *B. napus* and *A. thaliana*.

## Results

### Genome-wide identification of lncRNAs in *B. napus*

**Fig. 1:**
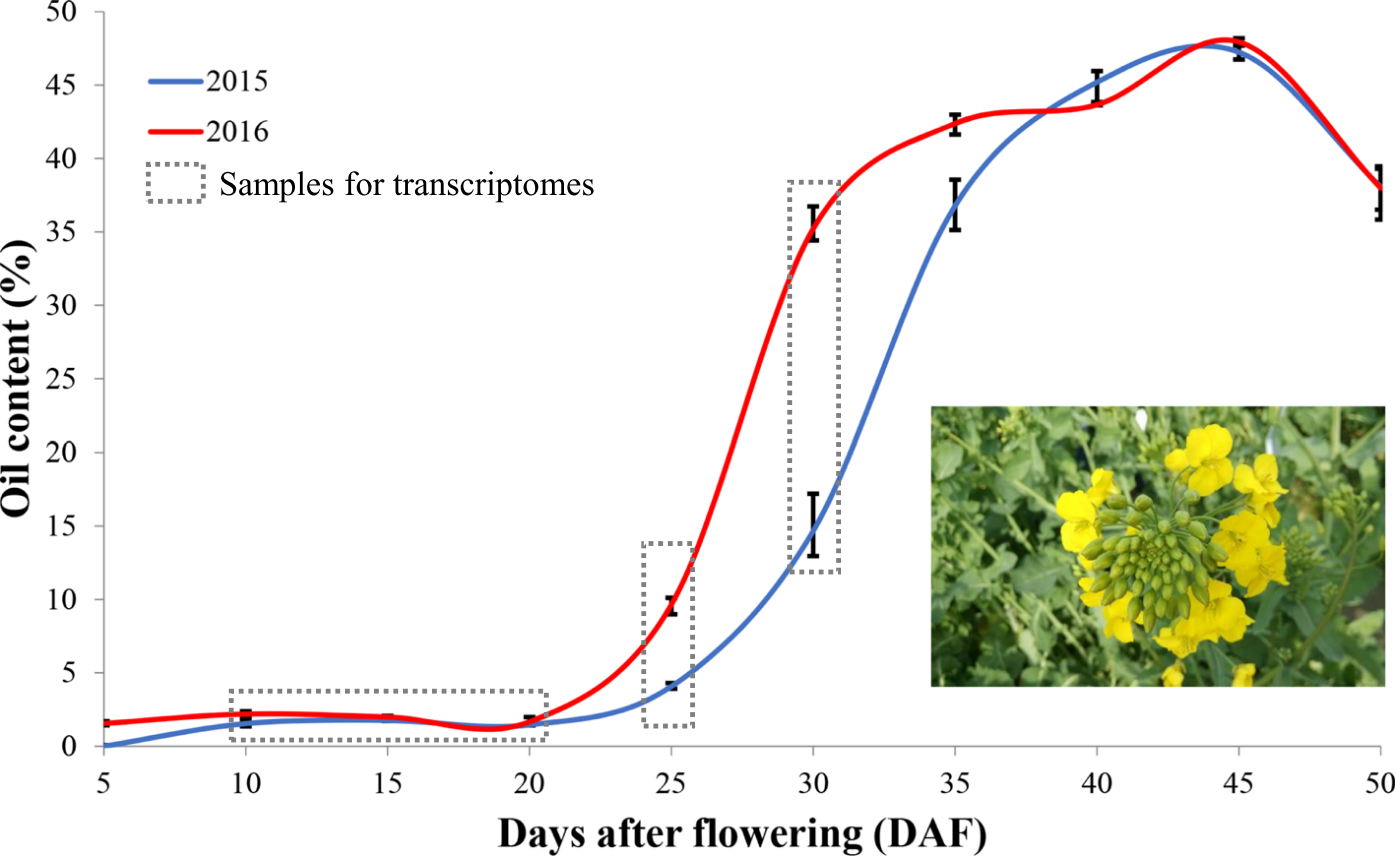
Oil content changes during seed development in *B. napus*. The X axis represents the days after flowering. The Y axis represents the oil content. Seeds from the time points indicated by dotted boxes were chosen for transcriptome analysis. Seeds from early developmental stages (10, 15 and 20 DAF) showed no change in oil content and were combined together to serve as a control in the transcriptome analysis

To identify lncRNAs related to lipid biosynthesis in *B. napus*, we first analyzed oil accumulation in developing seeds of the *B. napus* cultivar KenC-8. Developing seeds were collected from siliques every five days after flowering (DAF) up to 50 DAF (seed maturity) in two separate growing seasons. Little oil accumulation was observed in 5–20 DAF seeds, and the majority of oil accumulation occurred between 20 to 35 DAF. After 35–40 DAF, the rate of oil accumulation started to decrease (Fig. 1). Based on this oil accumulation profile, we selected four developmental stages for transcriptome analysis: 10–20 DAF (a developmental stage when little oil accumulation was occurring) was selected as baseline control and 25 DAF and 30 DAF were selected to represent two consecutive rapid oil accumulation stages. The RNAs from the developing seed samples of each growing season were kept and analyzed separately, whereas samples from the 10–20 DAF seeds were bulked (Fig. 1; Additional file 1: Table S1). In total, 20 (including two 40 DAF samples) ssRNA-seq libraries were created and sequenced, and more than 1.8 billion reads were generated for lncRNA identification. In addition we also collected 30 public transcriptomic datasets (including 14 from our previous study [45]) from poly(A) RNA-seq experiments using diverse tissues covering different periods of growth and development of *B. napus* (Additional file 1: Table S1).

**Fig. 2:**
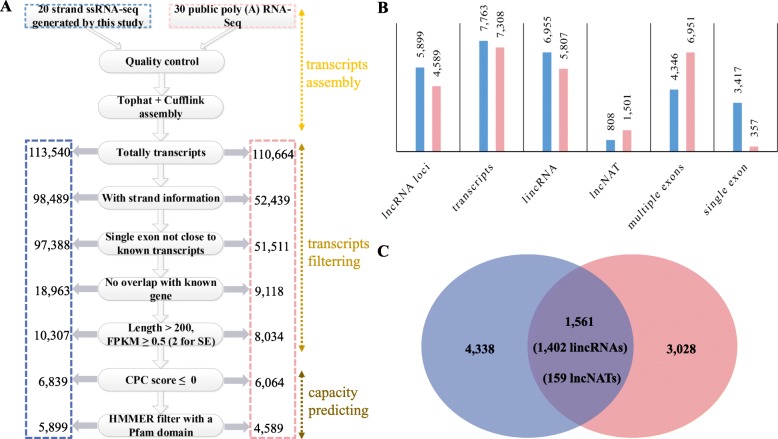
Identification of lncRNAs in *B.napus.* A, steps of the pipeline used in identification of lncRNAs from 20 rRNA removed strand specific RNA-seq (ssRNA-seq) datasets generated in this study and 30 poly(A) RNA-seq datasets downloaded from NCBI. B, number of lncRNA loci, transcripts, lincRNA, lncNAT and exons from the two types of RNA-seq datasets. C, comparison of lncRNAs identified in ssRNA-seq and poly(A) RNA-seq datasets. The blue color represents result from the 20 ssRNA-seq datasets; the pink color represents result from the 30 ploy (A) RNA-seq datasets

We adapted a previously established pipeline in rice [11] to process all transcriptome datasets. In brief, the pipeline cosists of three main steps, transcript assembly, filtering and protein-coding capacity prediction (Fig. 2a). The RNA-seq data was first mapped to the genome sequence of *B. napus* [39] to perform de novo transcript assembly. This step identified 113,540 and 110,664 transcripts in the ssRNA-seq and poly(A) RNA-seq datasets, respectively. Local perl scripts were then applied to filter out the transcripts that were shorter than 200 nucleotides, as well as transcripts with infrequent expression, without strand information, with single exon and very close to known transcripts, or overlapping with annotated genes. The final step was to estimate the coding potential for the remaining transcripts. In total, we identified 5899 (7763 transcripts) and 4589 lncRNA loci (7308 transcripts) from the ssRNA-seq and the ploy(A) RNA-seq datasets, respectively (Fig. 2a).

Combining results from the two datasets together, we identified 8905 non-redundant lncRNA loci, of which, 7100 were lincRNAs and 1805 were lncNATs (Additional file 2: Table S2). In total, 13,763 transcripts were identified from the 8905 non-redundant lncRNA loci, mainly due to alternative splicing events. The number of lincRNAs and lncNATs identified in the C_n_ subgenome was higher than that in the A_n_ subgenme (lincRNAs: 4130 versus 2763, 1.5 fold difference; lncNATs: 1076 versus 767, 1.4 fold difference; Additional file 3: Figure S1). This difference in complexity may be due to the differences in the size of the A_n_ (314.2 Mb) and C_n_ (525.8 Mb) genomes. Compared to the ssRNA-seq datasets (10.4%, 808 transcripts), the poly(A) RNA-seq datasets had a higher proportion (20.5%, 1501 transcripts) of lncNATs and a much higher proportion of single exon transcripts (44.0%, 3417 transcripts in the ssRNA-seq datasets versus to 4.9%, 357 transcripts in the poly(A) RNA-seq datasets) (Fig. 2b). Only about 20–30% of the lncRNA loci (1561, including 1402 lincRNAs and 159 lncNATs) were identified in both datasets (Fig. 2c), suggesting that, to have a full set of potential lncRNAs, it is necessary to use both library creating and sequencing methods in lncRNA identification.

### The properties of lncRNAs in allopolyploid *B. napus*

To gain a comprehensive understanding of the lincRNAs and lncNATs in *B. napus*, we compared several different features of the lincRNAs, lncNATs and mRNAs: exon numbers, transcript length, A/U content, relationship with transposable elements (TEs), and chromosome distribution.

**Fig. 3:**
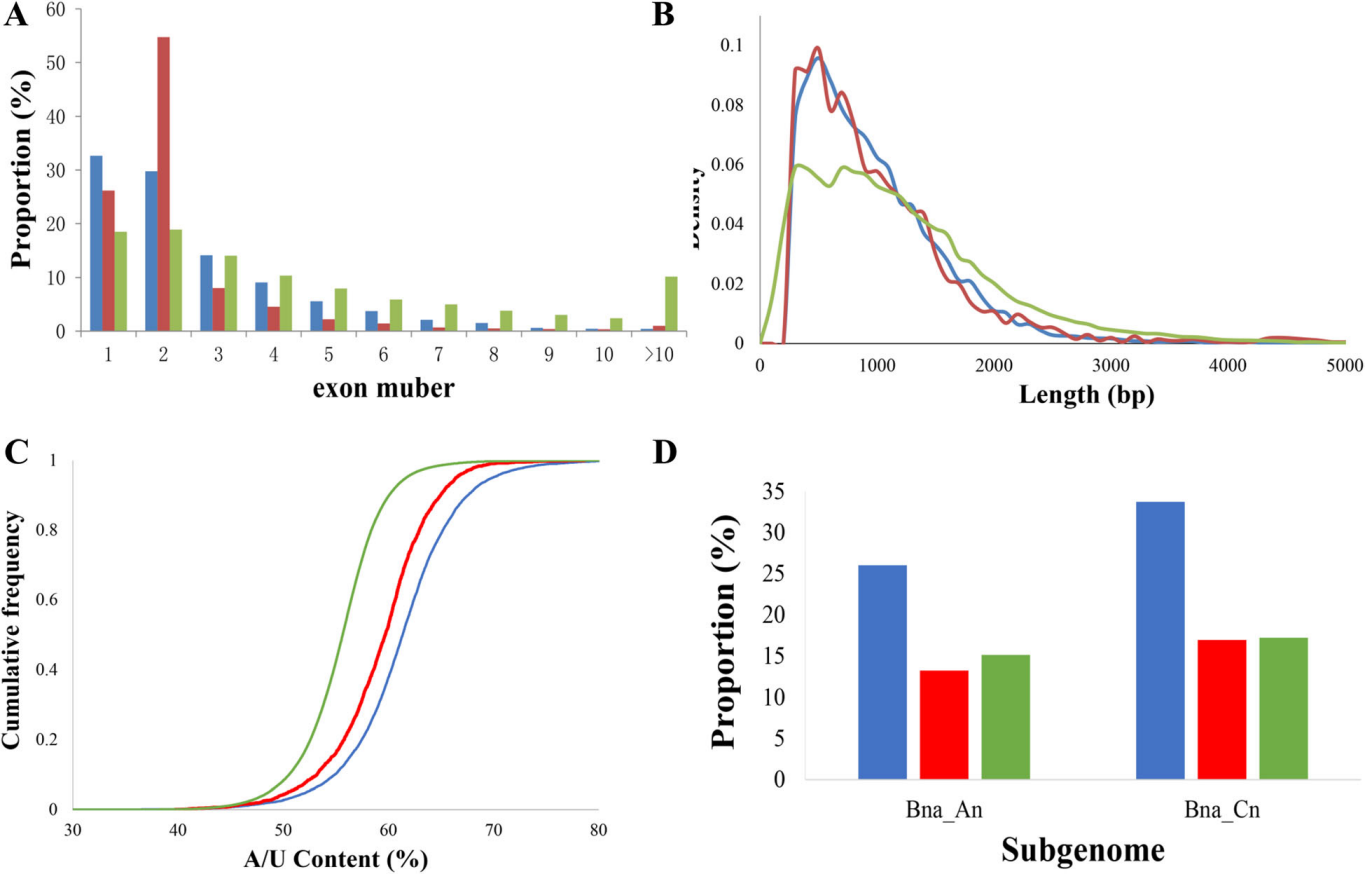
The properties of lncRNAs in *B. napus*. A, the distribution of lincRNA, lncNAT and mRNA with the exon number showing in the X-axis. B, the transcript length distribution of lincRNA, lncNAT and mRNA. C, A/U content of lincRNA, lncNAT and mRNA. D, the percentages of lincRNA, lncNAT and mRNA transcripts overlapping with transposable elements (TEs) in the A_n_ and C_n_ subgenomes. The blue, red and green colors represent lincRNAs, lncNATs and mRNAs, respectively

(1) With respespect to exon numbers, 32.7% and 29.8% of lincRNAs contained a single and two exons, respectively. The percentages of lncNATs containing a single and two exons were 26.2% and 54.7%, respectively. Both were much higher than those of protein coding transcripts (18.5% and 18.9%; Fig. 3a, Table 1). Most lincRNAs and lncNATs identified from the poly(A) RNA-seq datasets had two exons, while most lincRNAs and lncNATs identified from the ssRNA-seq datasets had a single exon (Additional file 4: Figure S2A, S2B). (2) Transcript length: The mean transcript length of the lncRNAs was 929 bp for lincRNAs and 985 bp for lncNATs. The transcript lengths of lncRNAs were obviously shorter than that of protein-coding genes (1287 bp; Fig. 3b, Table 1), although most transcripts of both lncRNAs and mRNAs are shorter than 2000 bp. The average lengths of lincRNAs and lncNATs were longer in the ssRNA-seq datasets than in the poly(A) RNA-seq datasets (lincRNAs: 967 bp vs 921 bp; lncNATs: 1168 bp vs 968 bp) (Additional file 4: Figure S2C, S2D). (3) When we analyzed A/U content we found that both the lincRNAs and lncNATs (particularly the lincRNAs), tended to be A/U-riched compared to protein coding sequences (Fig. 3c, Table 1). Transcripts with a high A/U content are less stable [46], suggesting that lncRNAs may be more flexible in interacting with other transcripts [47]. The A/U content difference between lincRNAs and lncNATs seemed to be smaller in the ssRNA-seq datasets than in the poly(A) RNA-seq datasets (Additional file 4: Figure S2E, S2F). (4) TEs account for 34.8% of the *B. napus* genome, with 25.9% in the A_n_ subgenome and 40.1% in the C_n_ subgenome [39]. When using ≥10 bp as an overlapping criterion, we found that 36.0% of lincRNAs were overlapping with TEs, with 32.2% and 38.2% in the A_n_ and the C_n_ subgenome, respectively. The proportion of lncNATs overlapping with TEs (13.3% in the A_n_ subgenome and 20.4% in the C_n_ subgenome) was close to that of mRNAs (15.2% in the A_n_ subgenome and 17.3% in the C_n_ subgenome) probably due to the co-localization of lncNATs with mRNAs (Fig. 3d, Table 1). Not surprisingly, we found that the proportion of lncNATs overlapping with TEs was much higher in the ssRNA-seq datasets than in the poly(A) RNA-seq datasets (Additional file 4: Figure S2G, S2H). (5) Of the lncRNAs that could be assigned to a chromosome location, the most (690 lncRNA loci, representing 523 lincRNAs and 167 lncNATs) were found to map to chromosome C03 and the least to chromosome A10 (201 lncRNA loci, representing 152 lincRNAs and 49 lncNATs) (Additional file 3: Figure S1).

**Table 1:** Comparison of the properties of lincRNA, lncNAT and mRNA in *B. napus*

Property	lincRNA	lncNAT	mRNA
Single exon (%)	32.7	26.2	18.5
Two exons (%)	29.8	54.7	18.9
Transcript length (bp)	929	985	1287
A/U content ranking	1st	2nd	3rd
TE in A_n_(%)	32.2	13.3	15.2
TE in C_n_(%)	38.2	20.4	17.3

### Coexpression analysis revealed potential function of lncRNAs in lipid biosynthesis

**Fig. 4:**
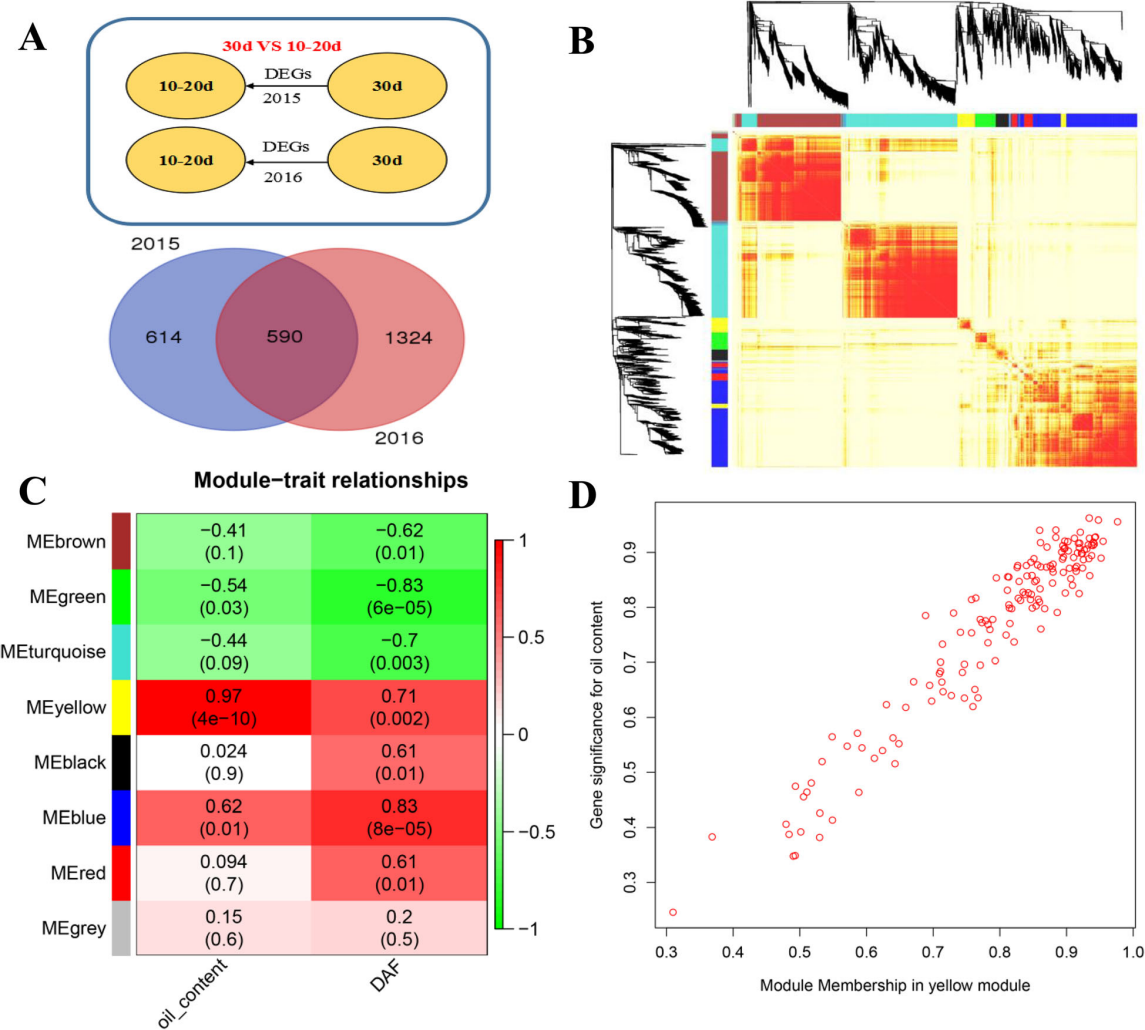
The coexpression analysis of lncRNAs between 30 DAF and 10–20 DAF in *B. napus*. A, the number of DEGs used in network construction in two years (2015 and 2016). B, hierarchical clustering with the topological overlap matrix to identify network modules consisting of the highly correlated genes. C, the correlation between each module and each of the two traits (oil content and DAF). D, the significance of each gene on oil content in the yellow module

**Fig. 5:**
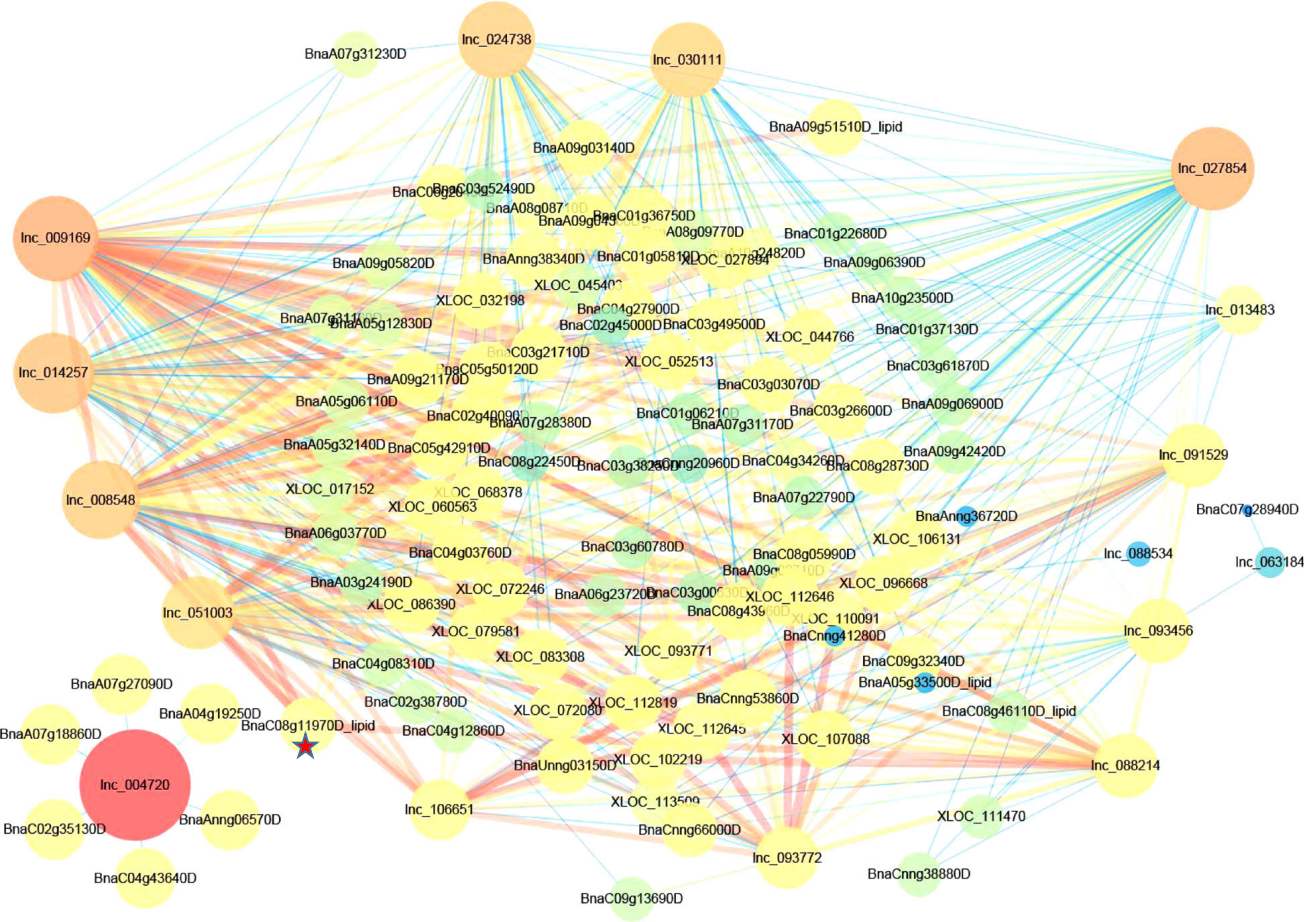
The network of lncRNAs and lipid-related genes based on the comparison of 30 vs 10–20 DAF in *B. napus*. A connection of the corresponding topological overlap was selected based on a threshold of 0.08. LncRNAs are shown in ‘lnc’ followed by a string of numbers. *B. napus* genes related to lipid metabolism are represented by gene ID_lipid. Newly predicted transcripts are named XLOC_number

It was previously reported that the *B. napus* genome contains ~ 2010 genes related to lipid biosynthesis [39]. To identify potential lncRNAs related to lipid biosynthesis, we applied Weighted Gene Coexpression Network Analysis (WGCNA) [48] to establish the coexpression network involving both protein coding genes predicted to be related to lipid biosynthesis and lncRNAs. We reasoned that such co-expressed lncRNAs would also be related to lipid biosynthesis. The analysis was done using protein coding genes and lncRNAs differentially expressed in the following three comparisons: 25 vs 10–20 DAF, 30 vs 10–20 DAF and 30 vs 25 DAF. We first identified differentially expressed lncRNAs and protein-coding genes in each comparison in each individual year, and then combined the DEGs (referring to both mRNAs and lncRNAs) from the 2 years in the coexpression analysis. In total, 1622 (including 104 lncRNA loci), 2528 (including 113 lncRNA loci) and 1416 (including 105 lncRNA loci) DEGs were identified in the three different developmental stages (Fig. 4a; Additional file 5: Figure S3A; Additional file 6: Figure S4a). A network was constructed for each comparison using the identified DEGs (Fig. 4b; Additional file 5: Figure S3B; Additional file 6: Figure S4B). The three networks (i.e., 25 vs 10–20 DAF, 30 vs 10–20 DAF and 30 vs 25 DAF) were partitioned into 9, 8 and 13 modules, respectively (Fig. 4c; Additional file 5: Figure S3C; Additional file 6: Figure S4C). The relationships between each module and the two traits (oil content and DAF) were computed. In the 25 vs 10–20 DAF comparison, only the green module was significantly correlated with both oil content and DAF (*p* < 0.01 and *p* < 2 * 10^− 4^; Additional file 5: Figure S3C). In the 30 vs 10–20 DAF comparison, the yellow and blue modules were significantly correlated with oil content and seven of the eight modules were significantly correlated with DAF (Fig. 4c). In the 30 vs 25 DAF comparison, the black module was the most significant module correlated with oil content (*p* < 2*10^− 8^) and the turquoise module was significantly correlated with both oil content and DAF (*p* < 0.002 and *p* < 1*10^− 4^; Additional file 6: Figure S4C). As examples, the significance of each individual gene is shown in the scatterplots for the three selected modules that showed the most significant correlation with oil-content in each comparison (Fig. 4d; Additional file 5: Figure S3D; Additional file 6: Figure S4D). We further applied Cytoscape [49] to display the lncRNA-related connections in the three modules (Fig. 5; Additional files 7 and 8: Figures S5, S6). Based on this analysis, 13 lncRNA loci were found to be correlated with 8 lipid-related genes (Additional file 9: Table S3). In the 25 vs 10–20 DAF comparison, two lncRNAs were co-expressed with three lipid-related genes, wheras in the 30 vs 10–20 DAF comparison, 10 lncRNAs were co-expressed with four lipid-related genes. Four lncRNAs were co-expressed with two lipid-related genes in the 30 vs 25 DAF comparison (Additional file 9: Table S3).

Among the eight lipid-related genes identified in our study was *BnaC08g11970D*, an ortholog of the Arabidopsis oleosin1 encoding gene *AT4G25140*. Oleosin is a protein found in oil bodies and involved in seed lipid accumulation. *BnaC08g11970D* is co-expressed with 9 lncRNA loci, including 8 in the 30 vs 10–20 DAF comparison and 4 in the 30 vs 25 DAF comparison. Three (lnc_008548, lnc_014257 and lnc_030111) of the 9 lncRNA loci were found to be co-expressed with *BnaC08g11970D* in both comparisons (Fig. 5; Additional file 8: Figure S6; Additional file 9: Table S3).

Among the other lipid biosynthesis related genes of note are *BnaC01g01840D*, *BnaA09g51510D* and *BnaC08g46110D*. *BnaC01g01840D* annotates as a patatin-related *phospholipase A* and is co-expressed with 4 lncRNAs. *BnaA09g51510D* and *BnaC08g46110D* may have roles in acetyl-CoA biosynthesis, and are co-expressed with 7 and 2 lncRNAs, respectively. *BnaC09g41580D* and *BnaA05g33500D* are predicted to encode one of the two ∆9 palmitoyl-ACP desaturases responsible for biosynthesis of *ω*-7 fatty acids in the maturing endosperm (Additional file 9: Table S3). The lncRNAs co-expressed with these two lipid-related genes may be involved in regulation of the expression of the lipid-related genes to play a role in lipid biosynthesis in *B. napus*.

**Fig. 6:**
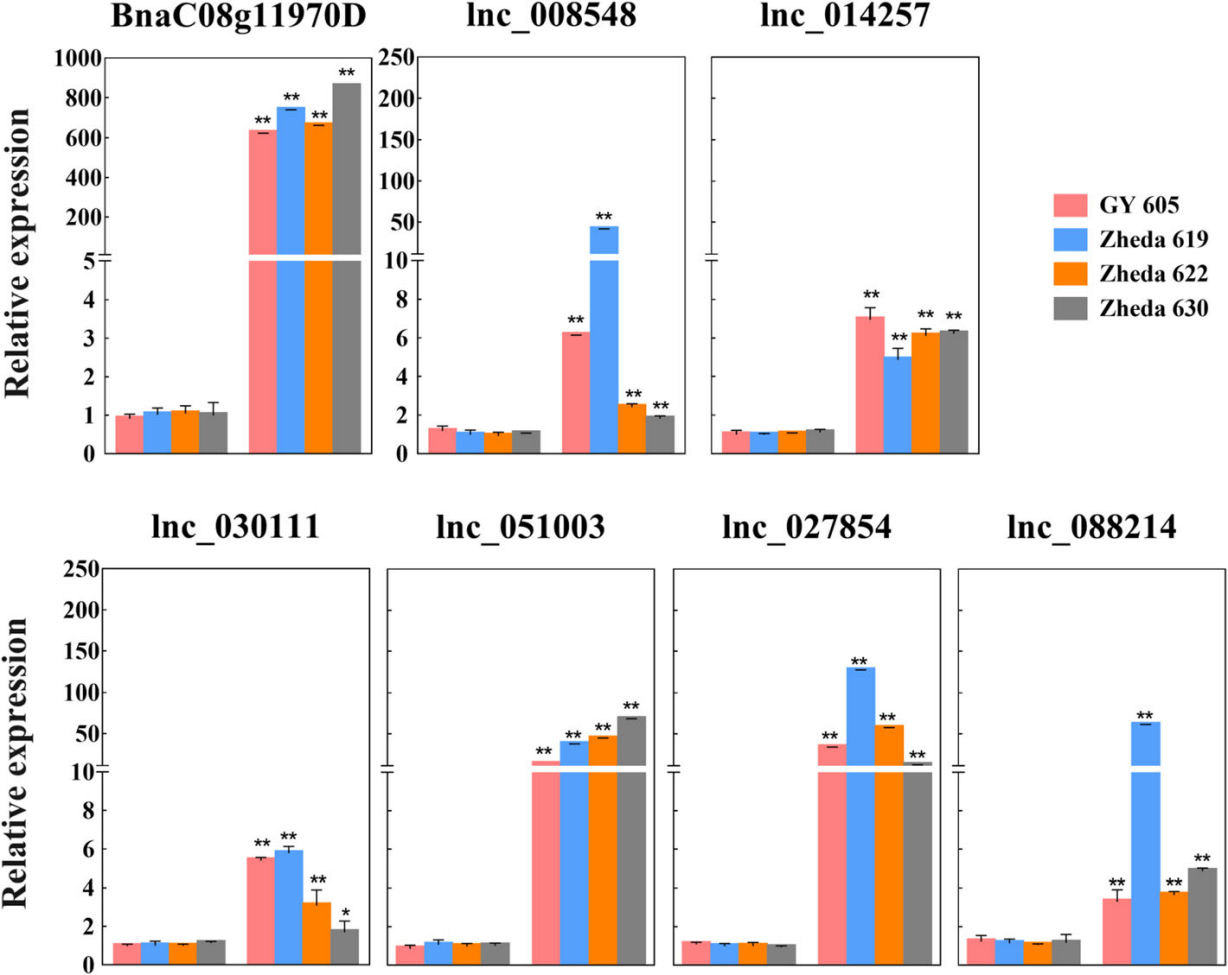
The relative expression levels of *BnaC08g11970D* and six co-expressed lncRNAs at the two developmental stages, 10–20 DAF and 30 DAF, in four oilseed cultivars (GY 605, Zheda 619, Zheda 622 and Zheda 630). For each graph, the left and right panels represent 10–20 DAF and 30 DAF, respectively

To verify the coexpression pattern of lncRNAs and lipid-related genes, we analyzed the expression changes of all 13 lncRNAs and 8 lipid-related genes at two developmental stages (10–20 DAF and 30 DAF) in four randomly selected oilseed cultivars, and were able to successfully generated results for 9 lncRNAs and 6 lipid-related genes. All 6 lipid-related genes (except *BnaA09g51510D* in GY605 and Zheda 622, and *BnaC09g41580D* in GY605) have a significantly higher expression level in 30 DAF seeds than in 10–20 DAF seeds in all four cultivars analyzed, particularly the two genes encoding putative oleosin1, *BnaC08g11970D* and *BnaC07g39370* (Fig. 6; Additional file 10: Figure S7). Consistent with the coexpression analysis results, the expression levels of all 9 lncRNAs were also significantly higher in 30 DAF seeds than in 10–20 DAF seeds in all four cultivars analyzed (Fig. 6; Additional file 10: Figure S7).

### Conservation of lncRNAs in *B. napus*

We estimated conservation of *B. napus* lncRNAs in other members (*B. rapa* and *B. oleracea* and *A. thaliana*) of the Brassicaceae family based on both sequence similarity and genomic synteny (details see Methods). For comparison of lncRNA sequences, we collected more than 40,000 previously identified lncRNAs in *A. thaliana* [19–22, 50], and identified 1259 and 1978 lncRNA loci in *B. rapa* and *B. oleracea,* respectively, using the publically available RNA-seq datasets (Additional file 11: Table S4; Additional file 12: Table S5). As shown in Tables 2 and Additional file 13: Table S6, only a small number of *B. napus* genomic sequences encoding lncRNAs had corresponding lncRNA sequences in *A. thaliana*, *B. oleracea* and *B. rapa* (316 (3.5%), 1074 (12.1%) and 731 (8.2%) respectively). Based on synteny analysis, the position of 3929 (44.1%), 2101 (23.6%) and 1729 (19.4%) lncRNA loci in *B. napus* were found to be conserved in *A. thaliana*, *B. oleracea* and *B. rapa*, respectively (supported by identified lncRNAs in the synteny regions; details in Additional file 14: Table S7). The sequences of most *B. napus* lncRNAs are not well conserved in *A. thaliana*, or in *B. oleracea* and *B. rapa*. Based on search against the whole genome sequences of *A. thaliana*, *B. oleracea* and *B. rapa*, only 809 (9.1%) *B. napus* lncRNAs were found to be conserved in *A. thaliana*, but 7476 (84.0%) and 7014 (78.8%) *B. napus* lncRNAs were found to be conserved in *B. oleracea* and *B. rapa*, respectively (Additional file 15: Table S8). These results suggest that *B. napus* lncRNAs have diverged significantly from *A. thaliana* but are well conserved in the closely related species. Low conservation of lncRNAs between *B. napus* and its two ancestors based on comparison of lncRNA sequences is probably because only a small portion of *B. oleracea* and *B. rapa* lncRNAs have been identified and used in comparison.

**Table 2:** Conservation of lncRNAs from *B. napus* (Bna) in *A. thaliana* (Ath), *B. oleracea* (Bol) and *B. rapa* (Bra)

Method	Comparison	Bna_An	Bna_Cn	Total^*^
Sequence-based^a^	Bna vs Ath	142	172	316 (2)
	Bna vs Bol	284	780	1074 (10)
	Bna vs Bra	441	284	731 (6)
Position-based^b^	Bna vs Ath	1767	2160	3929 (2)
	Bna vs Bol	763	1338	2101
	Bna vs Bra	822	907	1729

a: sequences with homologous coverage > 40%, E_value <1e-5 and identity > 50%;

b: at least one upstream and one downstream genes in the synteny region;

*: numbers in parentheses indicate the number of lncRNAs whose genomic location is undetermined

**Fig. 7:**
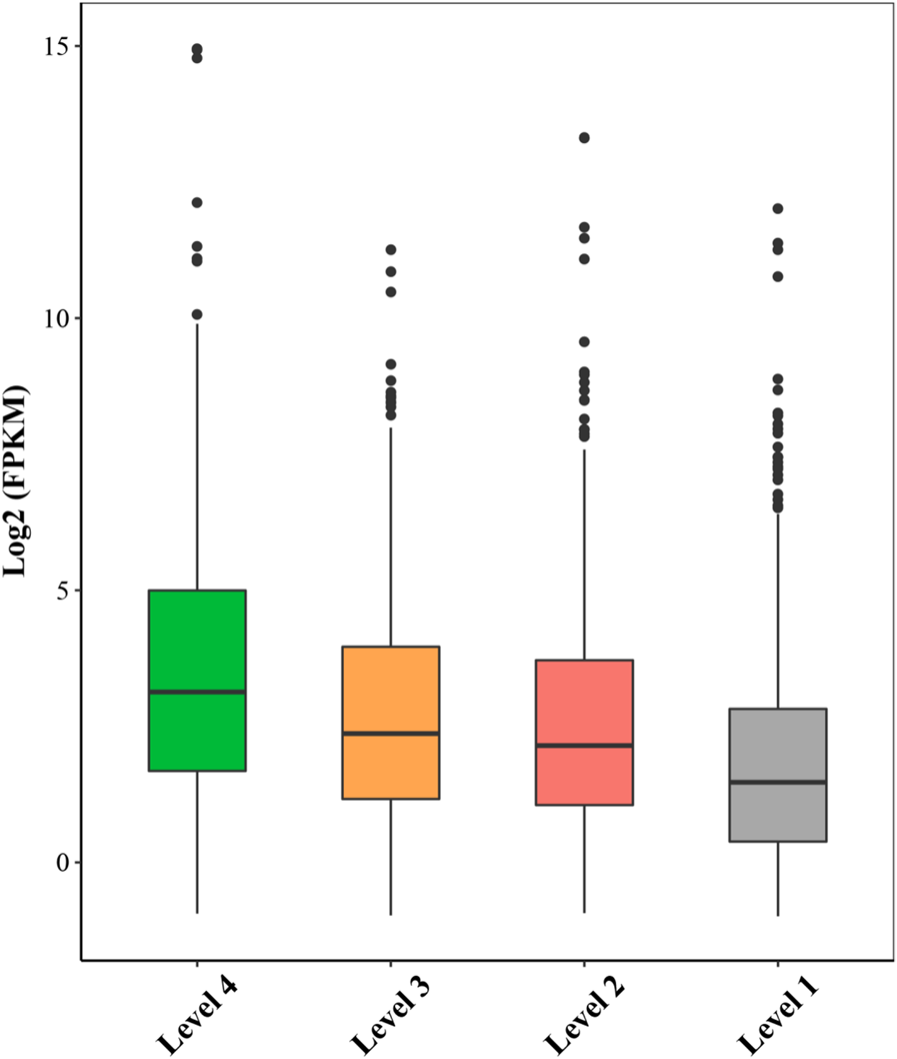
The expression levels of conserved lncRNAs among *B. napus*, *B. rapa*, *B. oleracea* and *A. thaliana*. The conserved *B. napus* lncRNAs were divided into four levels based on their homologous sequence coverage (Level 1: 20–40%; Level 2: 40–60%; Level 3: 60–80%; Level 4: 80–100%)

To study the relationship between the expression level and conservation of lncRNAs, we divided the *B. napus* lncRNAs conserved in other three species into four levels based on the coverage of homologous sequences (Level 1: 20–40%; Level 2: 40–60%; Level 3: 60–80%; Level 4: 80–100%). It seemed that the expression level of lncRNAs was positively correlated with the coverage of homologous sequences (Fig. 7). This is similar to the phenomenon observed in animals and human, where the evolutionary rate of the protein-coding genes and lincRNAs showed a negative correlation with expression level (i.e. highly expressed genes are on average more conserved during evolution [51]). For the 13 lncRNAs identified to be co-expressed with lipid-related mRNAs, none of them was conserved in *A. thaliana*, however, all of them had conserved sequences in *B. rapa* or *B. oleracea* at the genome level (Additional file 16: Table S9), suggesting that, at least in the Brassicaceae family, oil biosynthesis-related lncRNAs are lineage-specific.

## Discussion

Several studies have investigated the roles of small noncoding RNAs in lipid biosynthesis through small RNA sequencing and degradome sequencing [42–44], but no genome-wide study on lncRNAs has been previously carried out in *B. napus* up to now. In this study, we carried out the genome-wide study of lncRNAs in *B. napus* based on the newly sequenced *B. napus* genome, rRNA removed ssRNA-seq datasets generated from seeds of different developmental stages and publically available poly(A) RNA-seq datasets generated from diverse tissues. As a result, 7100 lincRNA loci and 1805 lncNAT loci were identified.

A large number of lncRNAs have been identified in many different plant species [11, 17, 19, 27, 32]. In Arabidopsis and rice, about half reported lncRNAs were un-spliced and contain only a single exon [11, 17, 19]. This feature was observed in *B. npaus* lncRNAs identified from rRNA-depleted total RNA, but not in the lncRNAs identified from poly(A) enriched mRNA (Additional file 4: Figure S2). Most *B. npaus* lncRNAs, particularly lncNATs, identified from the poly(A) enriched mRNA datasets contain two exons. Consequently, the average length of lncRNA transcripts (929 bp for lincRNAs and 985 bp for lncNATs) were longer in *B. napus* than in other plants. LncNATs had a higher proportion of multiple exons than lincRNAs (72% vs 60%). Compared to lncNATs, lincRNA are more likely to be overlapped with or derived from TEs, probably related to their genomic position. It seemed that TE-derived lncRNAs are more likely to generate alternative splicing events, compared to non-TE derived ones (18% vs 13%) (Additional file 17: Figure S8, Additional file 18: Table S10).

Two unique common features reported for lncRNAs are their low expression level and tissue-specific expression pattern [10, 11, 32]. Although we found the expression levels of both lincRNAs and lncNATs identified from the poly(A) RNA-seq datasets were lower than that of mRNAs (Additional file 19: Figure S9A), the expression levels of both lincRNAs and lncNATs identified from rRNA-depleted ssRNA-seq datasets were higher than that of mRNAs (Additional file 19: Figure S9B). Similar to *B. napus* homoeologous genes [39], on average, the A_n_ subgenome homoeologous lncRNAs seemed to have a higher expression level than the C_n_ subgenome ones (Additional file 20: Figure S10). In addition to the difference in exon numbers, lncRNAs identified from total RNA and mRNA also differ in their transcript length, A/U content, and degree of overlap with TEs (Additional file 4: Figure S2). These results together with the observed low level of overlap of the lncRNAs identified from total RNA and mRNA suggest that in order to capture a full set of lncRNAs and uncover as many features of the lncRNAs population as possible, it is necessary to use RNAs isolated from as diverse of a set of tissue and developmental staged samples as possible as a source of starting material.

Oil content is the most important agronomic trait of *B. napus* and increasing seed oil content is the final objective of many rapeseed breeding programs. Identifying genes involved in lipid biosynthesis regulation during seed development, including protein coding and non-coding ones, is an important first step towards improvement of the crop through genetic engineering. LncRNAs have been shown to play an important role in many aspects of plant development [15, 52–54]. Although it is now feasible to perform large scale lncRNA identification, it is still a challenge to study the function of lncRNAs and uncover the mechanism(s) underlying lncRNA-mediated regulation. Based on the rationale that genes involved in the same pathway(s) tend to be co-expressed, we reasoned that lncRNAs co-expressed with lipid-related genes would have a potential role in regulation of oil biosynthesis and accumulation in rapeseed. We found 13 lncRNAs whose expression patterns were significantly correlated with that of 8 lipid-related genes (Additional file 9: Table S3). Furthermore, these coexpression relationships were not related to the genomic location of the lncRNAs and lipid-related genes. Many of the coexpression relationships were further confirmed by qRT-PCR analysis of transcript levels in randomly selected *B. napus* cultivars. Among the coexpression modules, the relationships between several lncRNAs and *BnaC08g11970D* are particularly of interest. *BnaC08g11970D* is predicted to encode a protein homologous to oleosin1 of Arabidopsis, which contains a hydrophobic hairpin domain that is located in the surface of lipid droplets to make them stable and facilitate lipid accumulation [55]. The expression level of *BnaC08g11970D* is dramatically increased in the developmental stage of rapid seed oil accumulation (Figs. 1, 6), strongly suggesting a role of this gene in oil accumulation. LncRNAs co-expressed with this gene would thus be the ideal candidates of further studies to investigate their potential role(s) in regulating the expression and function of *BnaC08g11970D*. In summary, our finding point to the importance of examining the lncRNAs as a possible source of novel information and tools for *Brassica* improvement in the future.

## Methods

### Plant materials and generation of RNA-seq libraries

*Brassica napus* L. cv KenC-8 plants were grown in the field (Hangzhou, China) in 2015 and 2016. Flowers were tagged on the day of blooming (i.e. 0 day after flowering (DAF)). Every 5 days starting from 5 DAF and up to 50 DAF, seeds from 10 individual plants were harvested, pooled and used in oil content analysis. Based on the seed oil content change profile (Fig. 1), seeds from four developmental stages, i.e. early little oil accumulation (10–20 DAF), early rapid accumulation (25 DAF) and middle rapid accumulation (30 DAF) were used in transcriptome analysis. Two 40 DAF samples were also used in transcriptome analysis. Seeds harvested from these four stages were frozen immediately in liquid nitrogen and used in RNA extraction. Total RNA was isolated using BiooPure™ RNA Isolation Reagents and rRNA was removed by using the Ribo-Zero Kit (Epidemiology). RNA-seq libraries were constructed using the Illumina TruSeq Stranded RNA Kit and sequenced on the Illumina Hiseq 4000 (paired-end 150 bp).

### Public datasets used in this study

In total, we downloaded 45 publically available RNA-seq datasets from the National Center for Biotechnology Information (NCBI), including 30 poly(A) RNA-seq datasets from *B. napus* (accession number PRJEB5461, PRJEB2588, PRJNA262144, and PRJNA338132), 7 poly(A) RNA-seq datasets from *B. oleracea* (accession number PRJNA183713), and 8 poly(A) RNA-seq datasets from *B. rapa* (accession number PRJNA185152).

### Identification of lncRNAs

All of the raw reads from transcriptome sequencing were treated using Trimmomatic (Version 3.0) [56] with the default parameters for quality control. The clean data were then mapped to the *B. napus* genome using Tophat (Version 2.1.1) [57]. For each mapping result, Cufflinks (Version 2.1.1) [58] was used in transcript assembly. For strand-specific RNA-seq datasets, the parameter “--library-type fr-firststrand” was employed. All transcriptomes were merged with the annotated file from the reference genome to generate a final transcriptome using Cuffmerge. Cuffdiff was used to estimate the abundance of all transcripts based on the final merged transcriptome. We then used the following six filters to shortlist the bona fide lncRNAs from the obtained final transcriptome assembly: (1) transcripts without strand information were removed; (2) all single-exon transcripts that are within a 500-bp flanking region of known transcripts and in the same direction as the known transcripts were discarded; (3) transcripts overlapped with mRNAs annotated in the reference genome were deleted; (4) transcripts with FPKM scores < 0.5 (2 for single-exon transcripts) and shorter than 200 bp were discarded; (5) the coding potential value of each transcript was calculated using CPC [59] and those with CPC scores > 0 were discarded; (6) the remaining transcripts were searched against the Pfam database [60] by HMMER [61] to remove transcripts containing known protein domain. The transcripts remained were regarded as expressed candidate lncRNAs.

### Analysis of seed oil content

Seeds harvested at each developmental stage were dried in an incubator at 70 °C until their weight became stable. Isolation and GC analysis of seed lipids for total oil content and fatty acid compositions (expressed as μg/mg of total seed weight) were performed previously described [62, 63].

### The value of expression chosen for boxplot

The maximum FPKM of lncRNAs and mRNAs across all samples were selected as the expression values and used in generating of their expression distribution using Boxplot [10].

### Coexpression network construction

Weighted gene coexpression network analysis (WGCNA) [45] was used to predict the potential roles of lncRNAs in lipid biosynthesis. First, we defined a gene coexpression similarity by the Pearson correlation. Second, an adjacency function was employed to convert the coexpression similarity to connection strengths with a soft thresholding power in each comparison. Third, hierarchical clustering with the topological overlap matrix was used to identify network modules consisting of the highly correlated gene expression patterns. Finally, a summary profile (*eigengene*) for each module was used to correlate eigengenes with traits (oil content and DAF) and calculate the correlation between each gene and traits by defining Gene Significance (GS). The software Cytoscape was employed to visualize the networks [49].

### Positional synteny of lncRNAs

The synteny or co-linearity of lncRNAs among the four species (*B. napus*, *B. rapa*, *B. oleracea* and *A. thaliana*) was detected by MCScanX [64]. BLASTp was employed to determine the synteny by pairwise comparison with the parameters of E-value <1e-5 and max_target_seqs < 6. For each lncRNA, its 10 flanking protein coding loci were retrieved from the annotation of each genome. Homology tests of lncRNA and flanking genes among the four species were performed by BLASTn and the top 5 hits of each *B. napus* lncRNA were chosen for comparison of its flanking genes. A syntenic lncRNA pair among *B. napus*, *B. rapa*, *B. oleracea* and *A. thaliana* was defined by with at least one identical upstream or downstream flanking protein coding gene [42, 65].

### Sequence conservation of lncRNAs

To analyze the sequence conservation of lncRNAs, all the lncRNAs derived from *B. napus* were used as the query datasets and searched against lncRNAs from *B. rapa*, *B. oleracea* and *A. thaliana* and their genome sequences with BLASTn. The cutoff threshold for significant hits was an E-value <1e-5, coverage > 40% and identify > 50% for the matched regions [65].

### Quantitative reverse transcription (qRT)-PCR analysis

Total RNA isolated from seed samples of four cultivars at two stages 10–20 DAF and 30 DAF was used for first-strand cDNA synthesis using a HiScript II 1st Strand cDNA Synthesis kit (Vazyme) according to the manufacturer’s protocol. The cDNA was used as templates in qRT-PCR (ChamQ SYBR qPCR Master Mix-Q311 (Vazyme). Real-time PCR was performed using the LightCycler 96 (Roche). The reactions were performed at least in triplicate with three independent experiments, and the data were analyzed by the 2^-ΔΔct^ method. The primers used in our study were listed in Additional file 21: Table S11, including the reference gene (EF-1α). All values are presented as fold changes of 30 DAF to 10–20 DAF. Student’s t-test was performed to determine significant changes (*P* < 0.05).

## Conclusions

In this study, a total of 8905 lncRNA loci were identified, including 7100 lincRNA loci and 1805 loci generating lncNAT. We demonstrated that the *B. napus* genome has a large number of lncRNA and that these lncRNAs are expressed broadly across many developmental times and in different tissue types. We also provide evidence indicating that specific lncRNAs appear to be important regulators of lipid biosynthesis forming regulatory networks with transcripts involved in lipid biosynthesis. We also provide evidence that these lncRNAs are conserved in other species of the Brassicaceae family. Taken together, our data will provide insight into the further study of lncRNAs roles in oil biosynthesis in *B.napus*.

## Additional files

Additional file 1: **Table S1.** The RNA-seq datasets used in this study. (XLS 28 kb)

Additional file 2: **Table S2.** The detail information of lncRNAs identified in *B. napus*. (XLS 3981 kb)

Additional file 3: **Figure S1.** The chromosomal distribution of *B. napus* lncRNAs. (PPT 207 kb)

Additional file 4: **Figure S2.** The comparisons of lncRNA properties between the two sequencing methods. (PPT 789 kb)

Additional file 5: **Figure S3.** The coexpression analysis of *B. napus* lncRNAs between 25 DAF and 10–20 DAF*. (PPT 3838 kb)*

Additional file 6: **Figure S4.** The coexpression analysis of *B. napus* lncRNAs between 30 DAF and 25 DAF. (PPT 4123 kb)

Additional file 7: **Figure S5.** The network of the *B. napus* lncRNAs and their connected genes in the green module under the comparison of 25 DAF versus 10–20 DAF. (PPT 380 kb)

Additional file 8: **Figure S6.** The network of the *B. napus* lncRNAs and their connected genes in the black module under the comparison of 30 DAF versus 25 DAF. (PPT 873 kb)

Additional file 9: **Table S3.** The lncRNAs correlated with lipid-related genes detected by WGCNA. (XLS 24 kb)

Additional file 10: **Figure S7.** The relative expression levels of 5 lipid genes and six lncRNAs at the two developmental stages, 10–20 DAF and 30 DAF, in the four oilseed cultivars (GY 605, Zheda 619, Zheda 622 and Zheda 630). In each gene and lncRNA, the left panel represents 10–20 DAF, and the right panel represents 30 DAF. (PPT 1206 kb)

Additional file 11: **Table S4.** The detail information of lncRNAs identified in *B. rapa*. (XLS 462 kb)

Additional file 12: **Table S5.** The detail information of lncRNAs identified in *B. oleracea*. (XLS 765 kb)

Additional file 13: **Table S6.** The sequence conservation of *B. napus* lncRNAs in *B. rapa*, *B. oleracea* and *A. thaliana* based on lncRNAs identified in the three species. (XLSX 200 kb)

Additional file 14: **Table S7.** The positional conservation of in *B. napus* lncRNAs *B. rapa*, *B. oleracea* and *A. thaliana* based on lncRNAs identified in the tree species. (XLSX 4524 kb)

Additional file 15: **Table S8.** The genome sequence based conservation of *B. napus* lncRNAs in *B. rapa*, *B. oleracea* and *A. thaliana*. (XLSX 951 kb)

Additional file 16: **Table S9.** The genome sequence based conservation of 13 lipid-related *B. napus* lncRNA loci in *B. rapa*, *B. oleracea* and *A. thaliana*. (XLS 20 kb)

Additional file 17: **Figure S8.** The proportion of one or multiple exons, TEs and alternative splicing in lncRNAs and lncNATs. (PPT 143 kb)

Additional file 18: **Table S10.** The proportion of one or multiple exons, TEs and alternative splicing in lncRNAs and lncNATs. (XLSX 32 kb)

Additional file 19: **Figure S9.** Boxplot showing the distribution of maximum FPKM of lincRNAs, lncNATs and mRNAs across all samples. (PPT 219 kb)

Additional file 20: **Figure S10.** The expression levels of homoeologous lncRNAs in the two subgenomes in *B.napus*. (PPT 102 kb)

Additional file 21: **Table S11.** The primers used in this study. (XLSX 10 kb)
