# Supplementary figures and images for: Genome-wide identification of oil biosynthesis-related long non-coding RNAs in allopolyploid Brassica napus
Source: BMC Genomics. 2018 Oct 12;19:745. doi: 10.1186/s12864-018-5117-8 (PMC6186049; doi:10.1186/s12864-018-5117-8)

## Slide 1
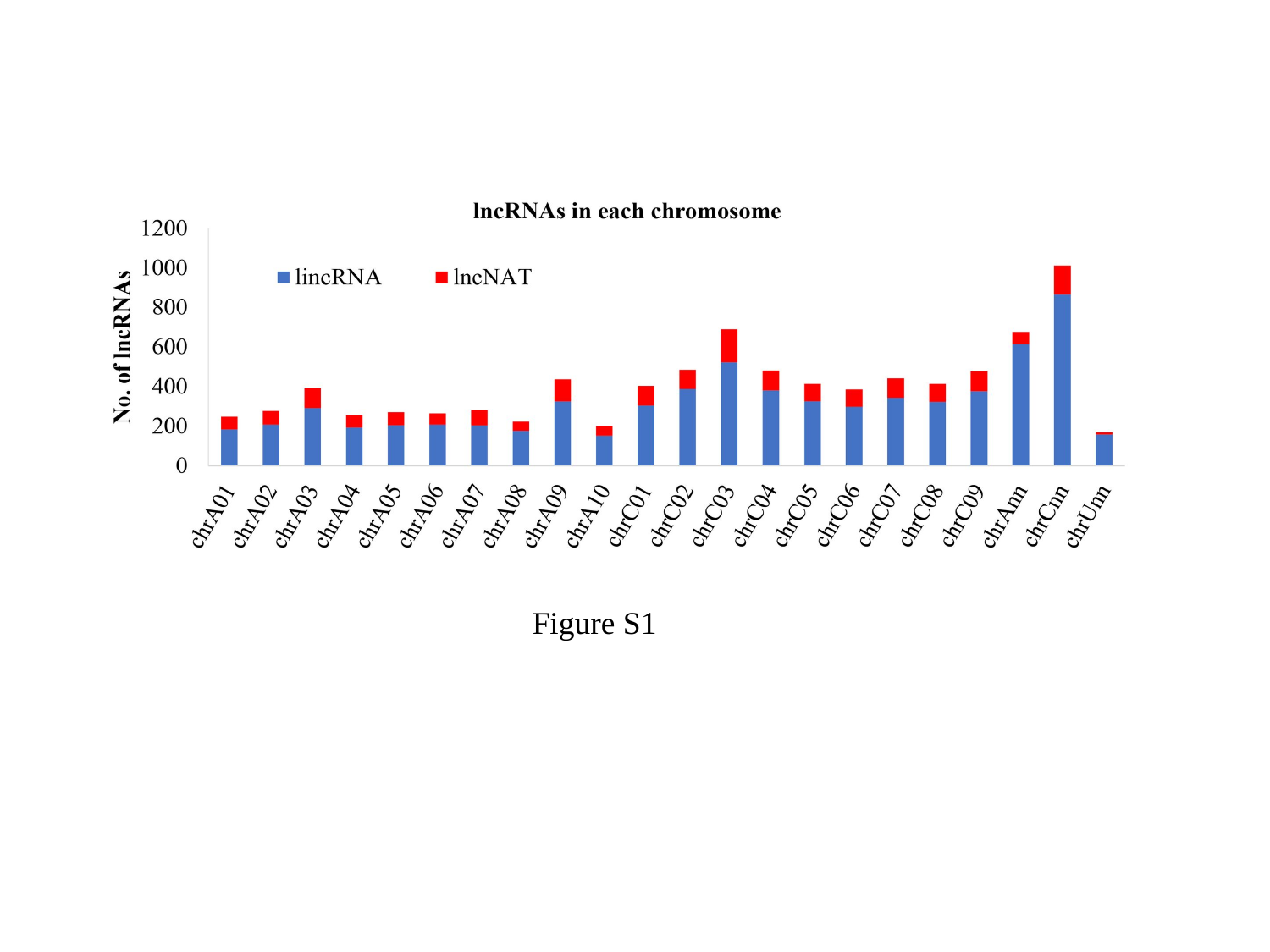

Figure S1

Supplement: Supplementary file 3 — Figure S1. The chromosomal distribution of B. napus lncRNAs. (PPT 207 kb) [file 12864_2018_5117_MOESM3_ESM.ppt]

## Slide 1
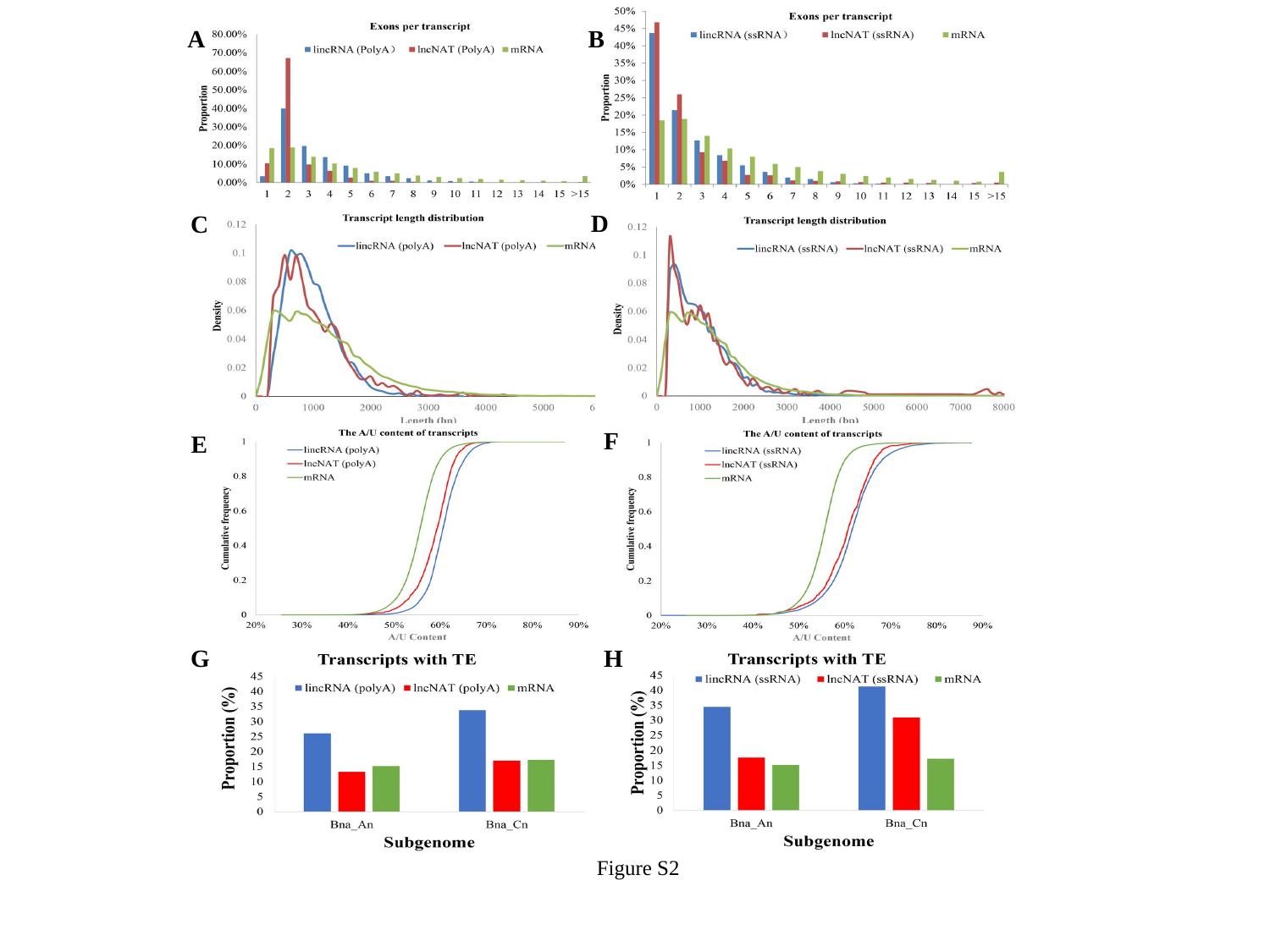

B
A
D
C
F
E
G
H
Figure S2

Supplement: Supplementary file 4 — Figure S2. The comparisons of lncRNA properties between the two sequencing methods. (PPT 789 kb) [file 12864_2018_5117_MOESM4_ESM.ppt]

## Slide 1
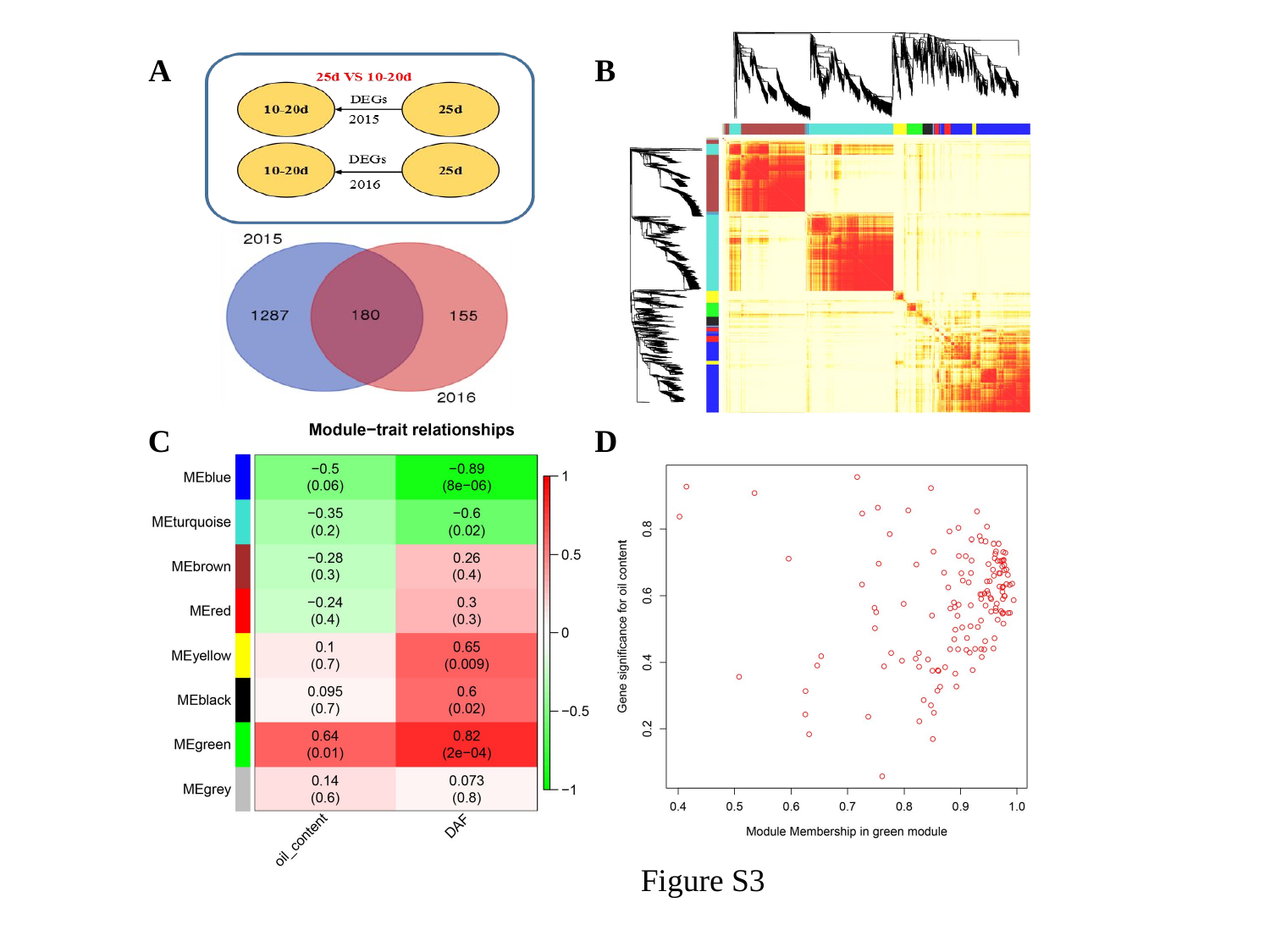

A
B
C
D
Figure S3

Supplement: Supplementary file 5 — Figure S3. The coexpression analysis of B. napus lncRNAs between 25 DAF and 10–20 DAF. (PPT 3838 kb) [file 12864_2018_5117_MOESM5_ESM.ppt]

## Slide 1
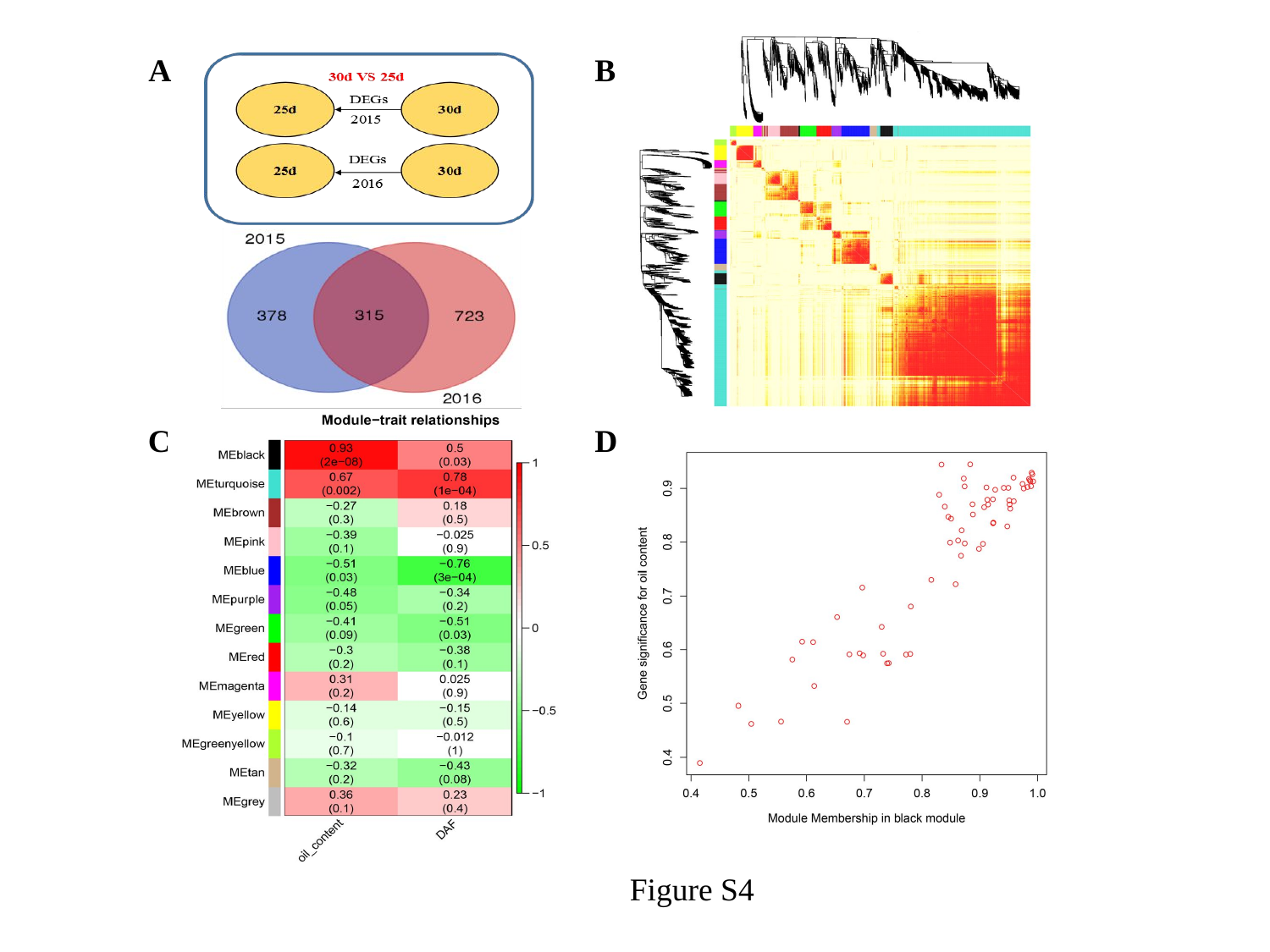

A
B
C
D
Figure S4

Supplement: Supplementary file 6 — Figure S4. The coexpression analysis of B. napus lncRNAs between 30 DAF and 25 DAF. (PPT 4123 kb) [file 12864_2018_5117_MOESM6_ESM.ppt]

## Slide 1
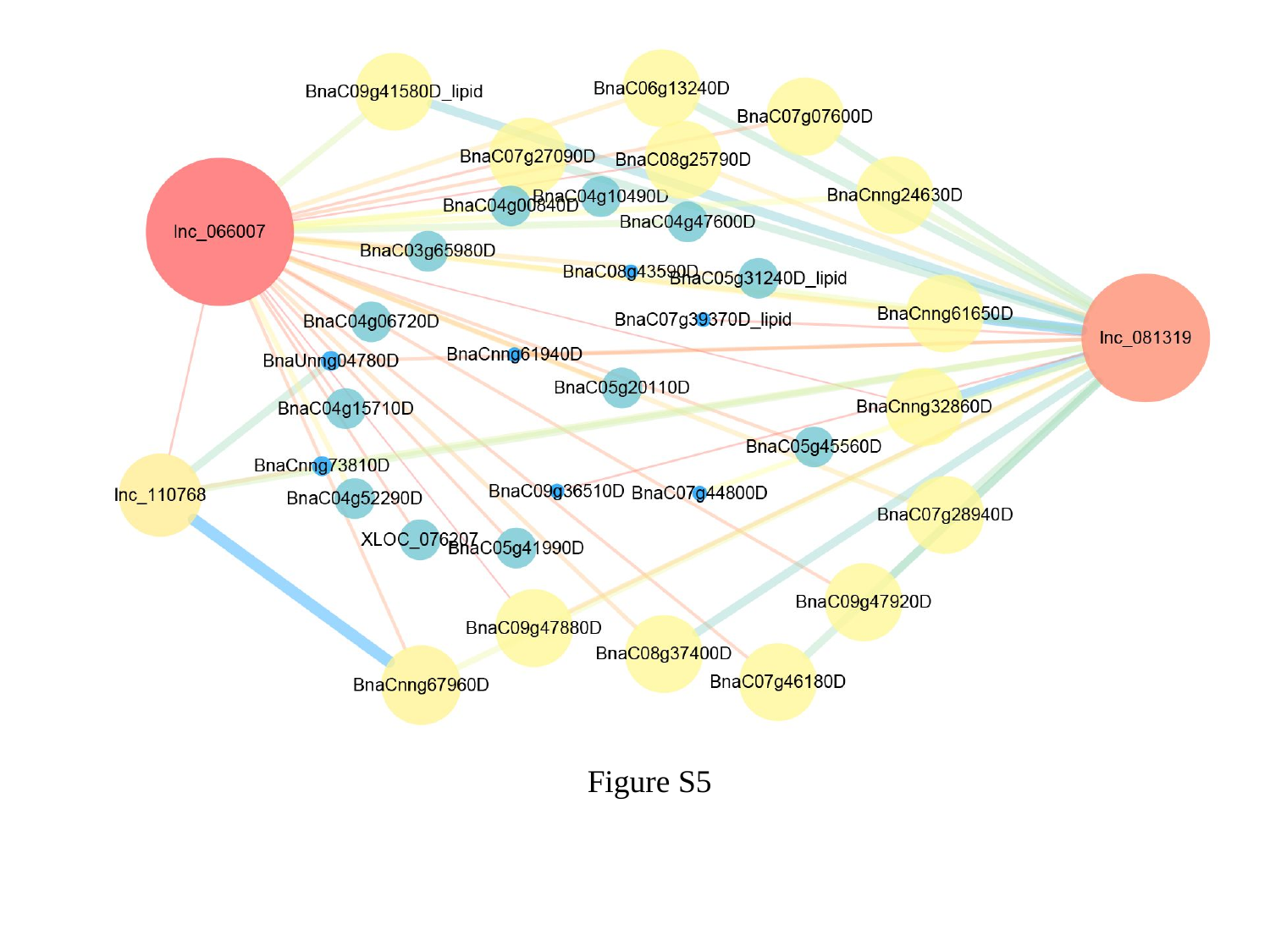

Figure S5

Supplement: Supplementary file 7 — Figure S5. The network of the B. napus lncRNAs and their connected genes in the green module under the comparison of 25 DAF versus 10–20 DAF. (PPT 380 kb) [file 12864_2018_5117_MOESM7_ESM.ppt]

## Slide 1
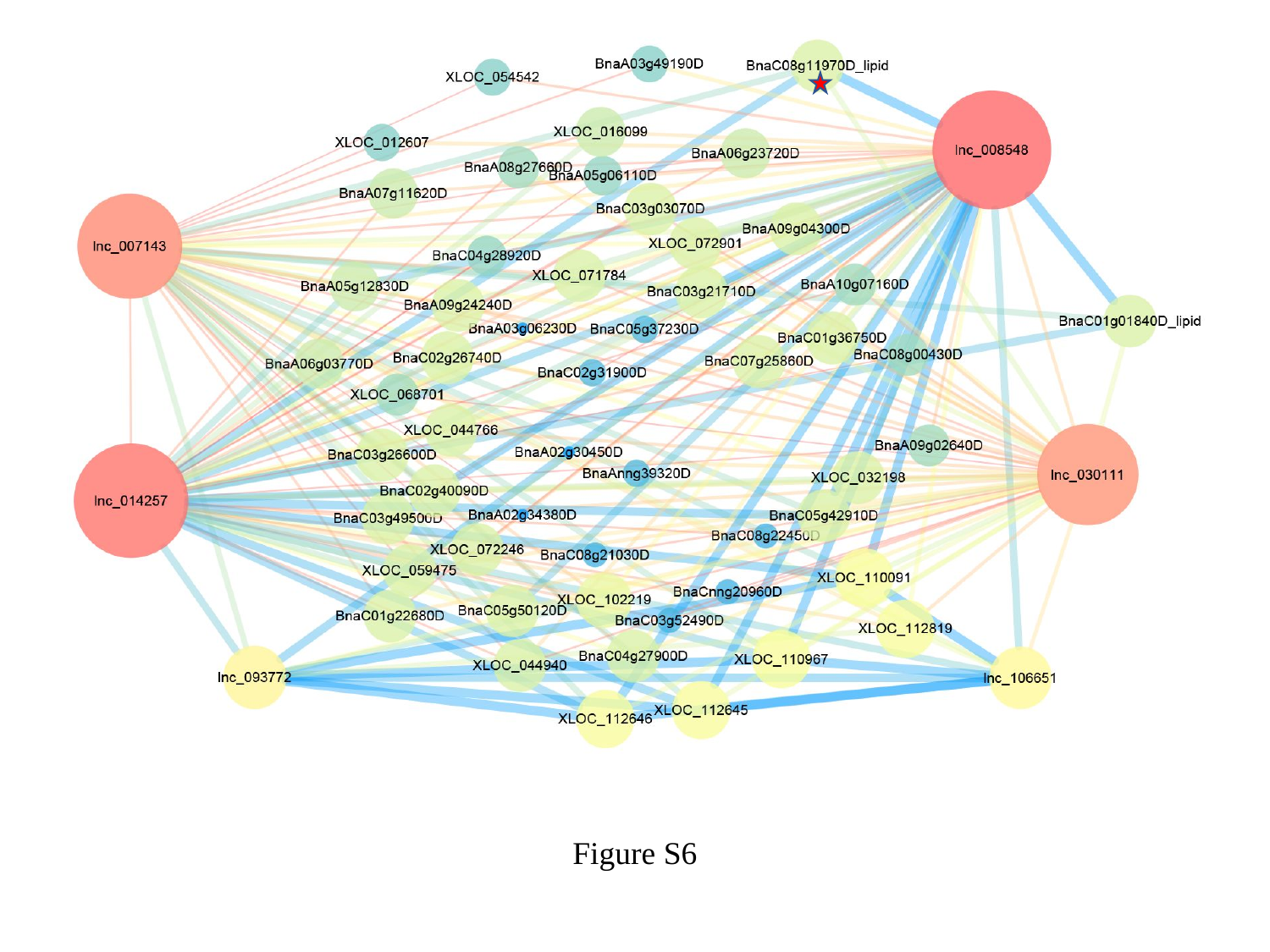

Figure S6

Supplement: Supplementary file 8 — Figure S6. The network of the B. napus lncRNAs and their connected genes in the black module under the comparison of 30 DAF versus 25 DAF. (PPT 873 kb) [file 12864_2018_5117_MOESM8_ESM.ppt]

## Slide 1
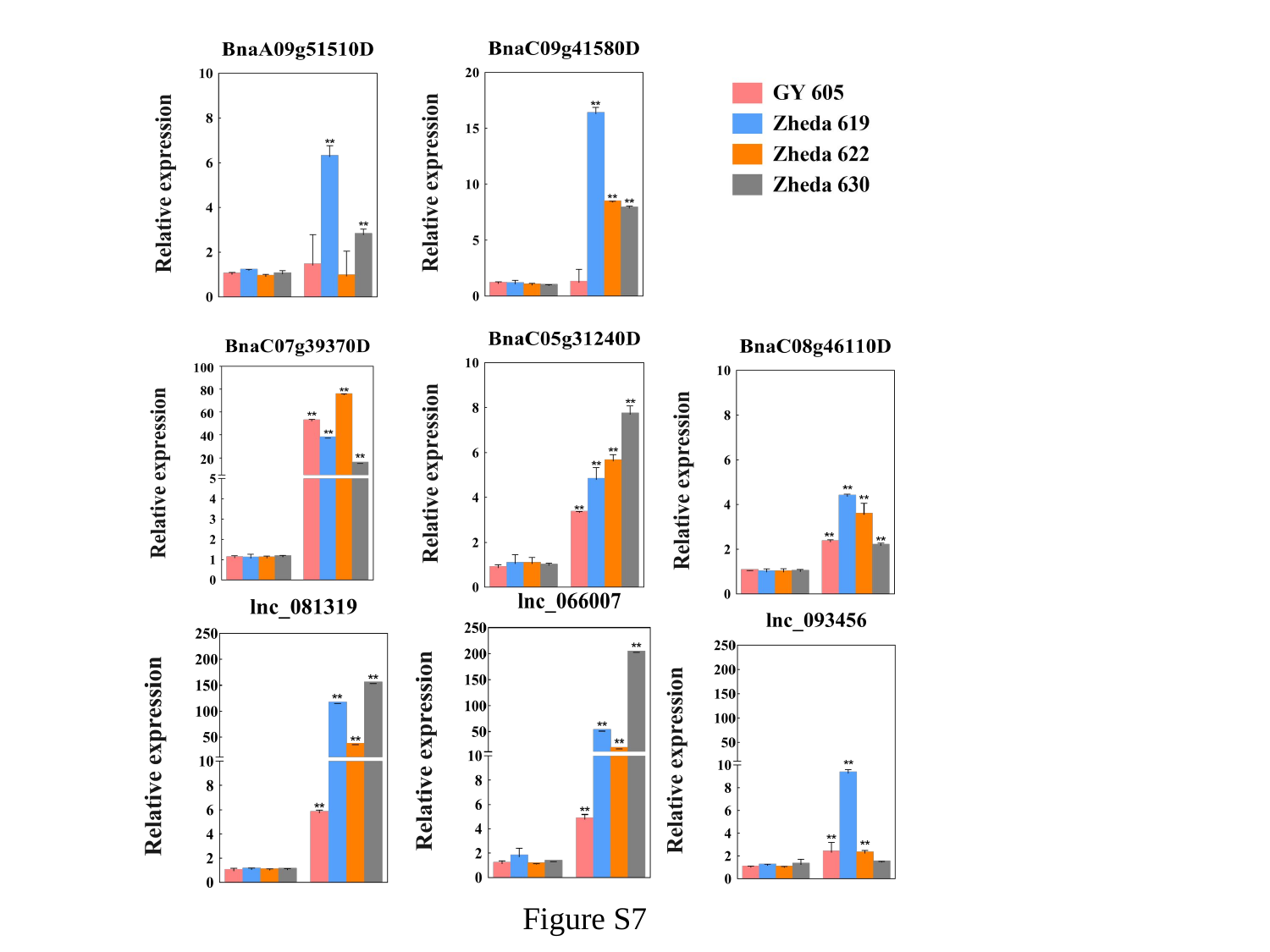

Figure S7

Supplement: Supplementary file 10 — Figure S7. The relative expression levels of 5 lipid genes and six lncRNAs at the two developmental stages, 10–20 DAF and 30 DAF, in the four oilseed cultivars (GY 605, Zheda 619, Zheda 622 and Zheda 630). In each gene and lncRNA, the left panel represents 10–20 DAF, and the right panel represents 30 DAF. (PPT 1206 kb) [file 12864_2018_5117_MOESM10_ESM.ppt]

## Slide 1
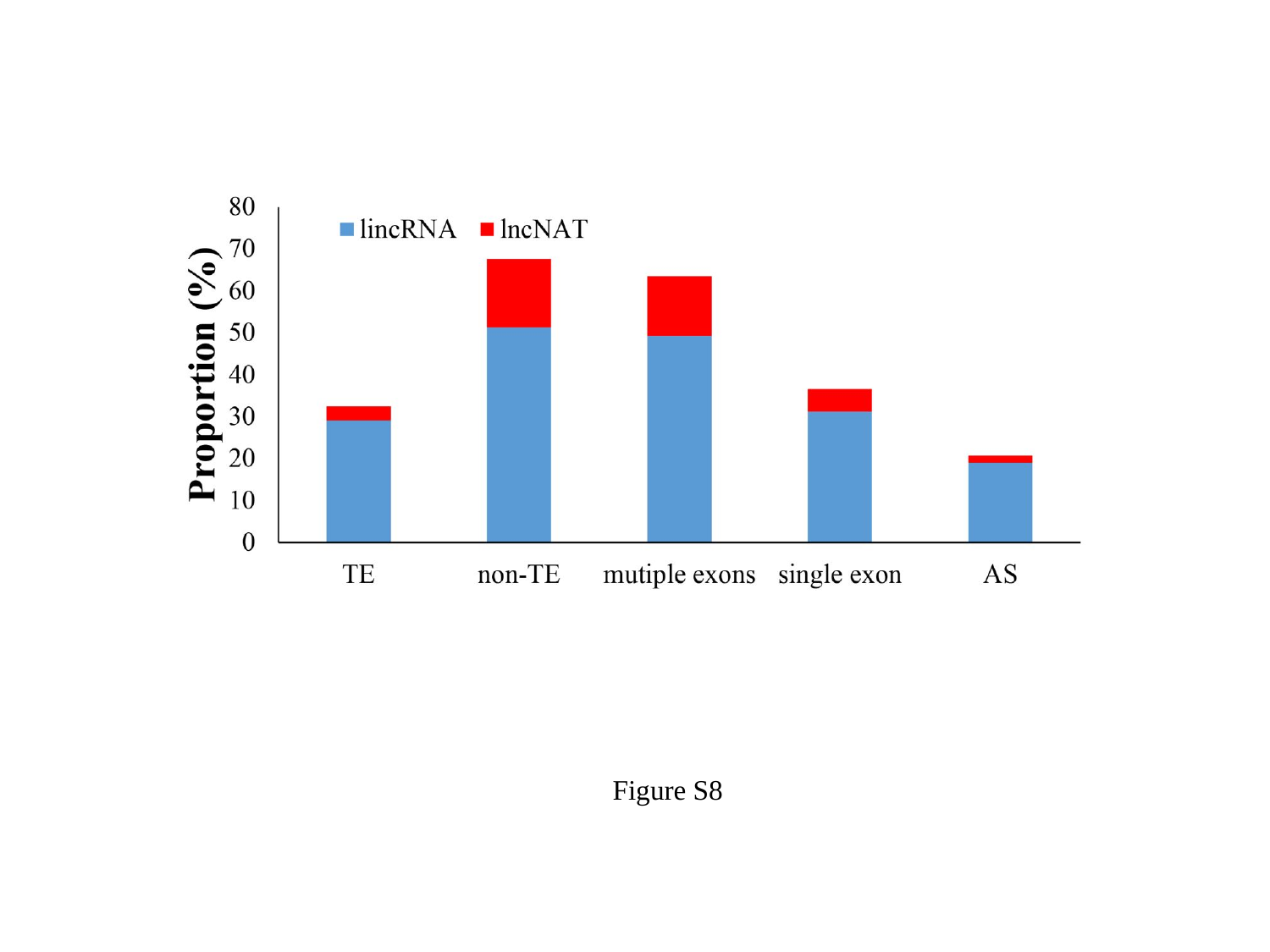

Figure S8

Supplement: Supplementary file 17 — Figure S8. The proportion of one or multiple exons, TEs and alternative splicing in lncRNAs and lncNATs. (PPT 143 kb) [file 12864_2018_5117_MOESM17_ESM.ppt]

## Slide 1
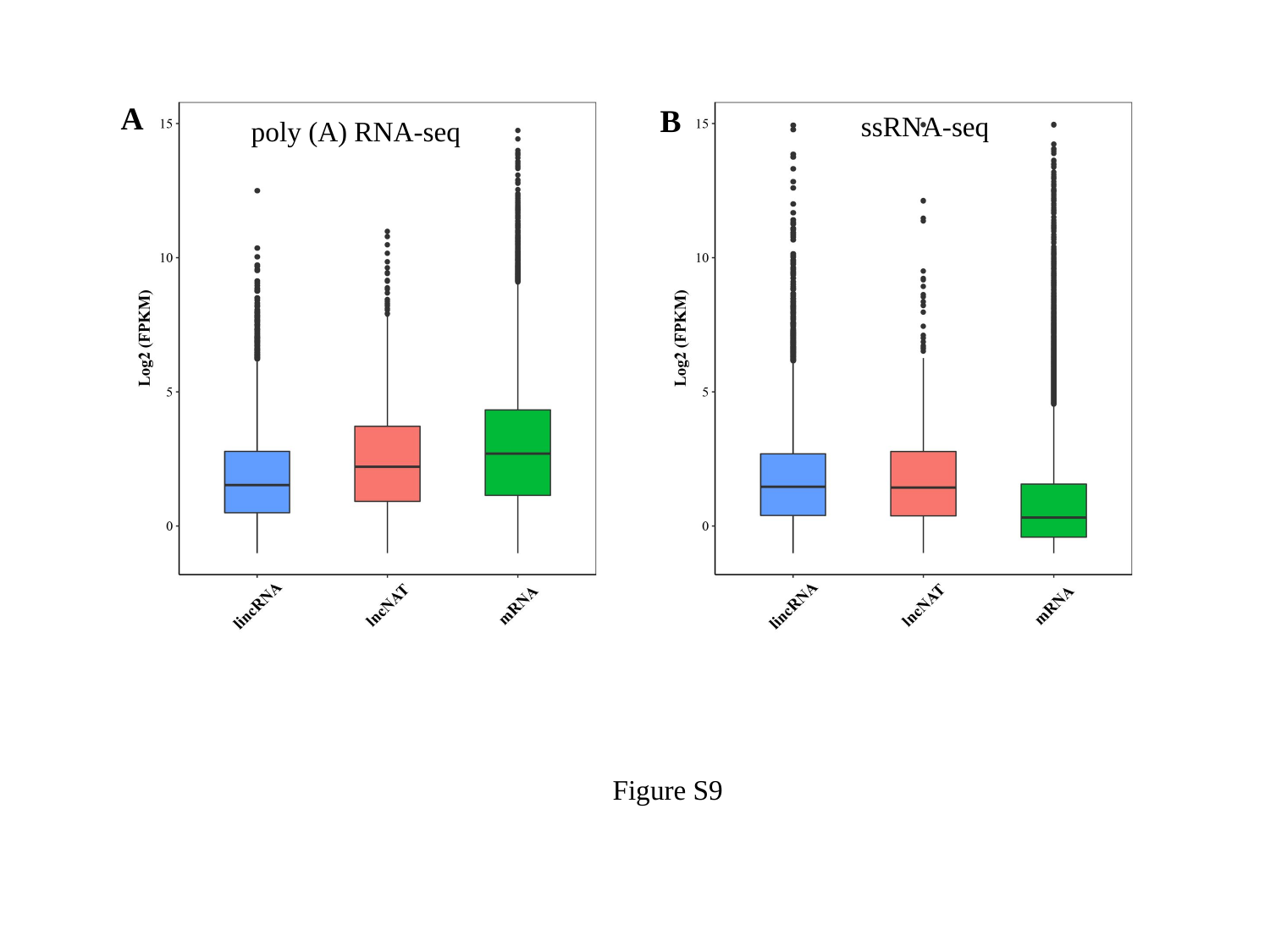

A
B
ssRNA-seq
poly (A) RNA-seq
Figure S9

Supplement: Supplementary file 19 — Figure S9. Boxplot showing the distribution of maximum FPKM of lincRNAs, lncNATs and mRNAs across all samples. (PPT 219 kb) [file 12864_2018_5117_MOESM19_ESM.ppt]

## Slide 1
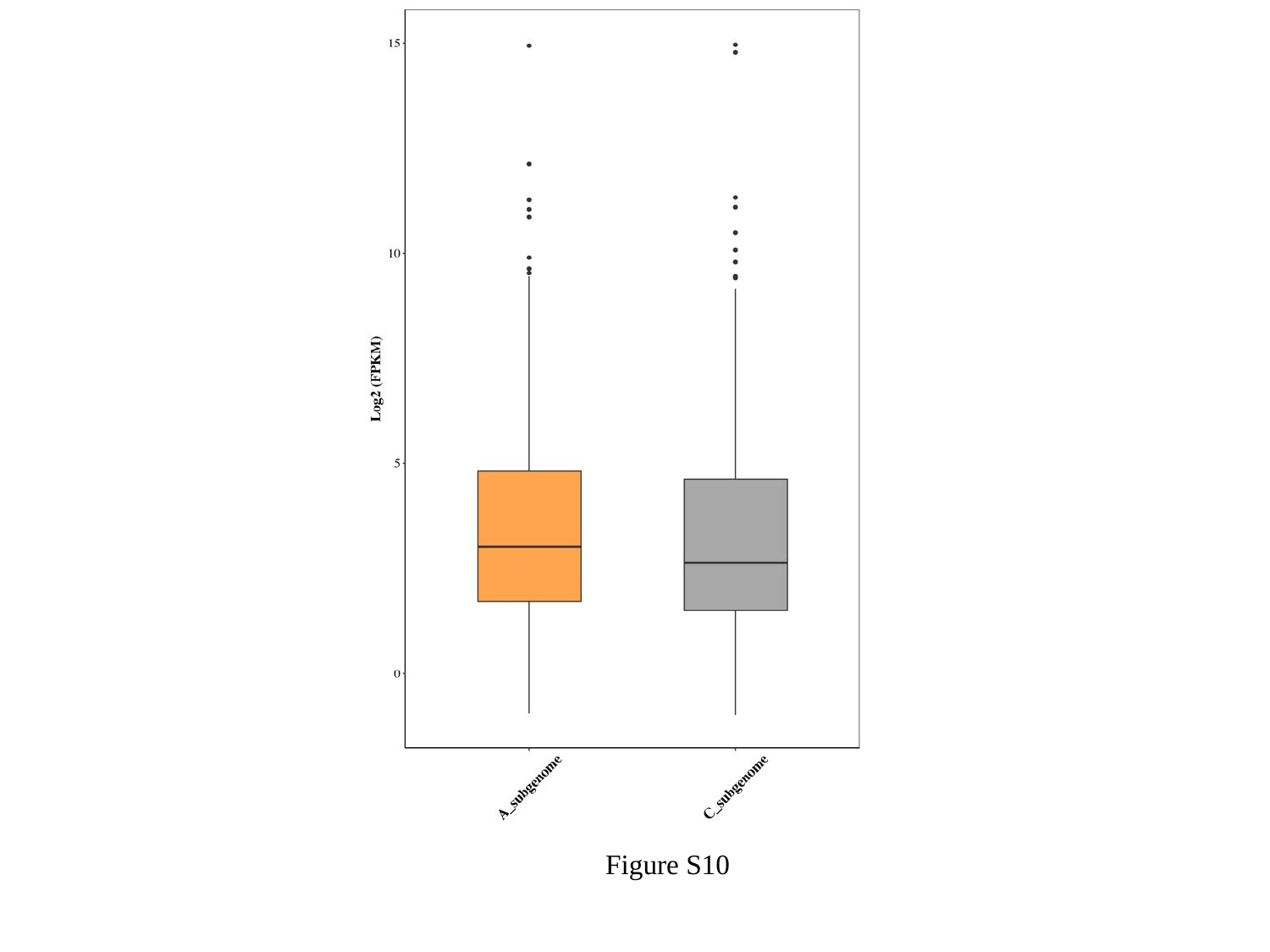

Figure S10

Supplement: Supplementary file 20 — Figure S10. The expression levels of homoeologous lncRNAs in the two subgenomes in B.napus. (PPT 102 kb) [file 12864_2018_5117_MOESM20_ESM.ppt]
